# A mixed methods study protocol evaluating early screening, triaging, risk assessment and health optimisation in perioperative pathways

**DOI:** 10.1371/journal.pone.0335129

**Published:** 2025-11-05

**Authors:** Katie Gilchrist, Bo Hou, James Bedford, Patrick Nyikavaranda, Margaret Ogden, Grainne Brady, Angus I. G. Ramsay, Ramani Moonesinghe, Snehal M. Pinto Pereira, Cecilia Vindrola-Padros

**Affiliations:** 1 Research Department of Targeted Intervention, Division of Surgery and Interventional Science, Central London Patient Safety Research Collaboration (PSRC), University College London, London, United Kingdom; 2 UCLH Surgical Outcomes Research Centre, Department of Anaesthesia and Peri-operative Medicine, University College London Hospitals NHS Foundation Trust, London, United Kingdom; 3 Department of Primary Care and Public Health, Brighton & Sussex Medical School, University of Sussex, United Kingdom; 4 Division of Psychiatry, Faculty of Brain Sciences, University College London, United Kingdom,; 5 Department of Behavioural Science and Health, University College London, London, United Kingdom; IRCCS: IRCCS Ospedale San Raffaele, ITALY

## Abstract

**Background:**

Over 10 million operations are carried out every year in the UK, improving millions of lives. While most operations are low-risk, some result in patients having poor outcomes. Preoperative assessment evaluates a patient’s health prior to surgery to identify risks and where possible minimise them through optimisation. However, this preoperative assessment often takes place close to the planned date of surgery, meaning there is little time to optimise patients’ health. Early preoperative screening is the process of evaluating a patient’s health earlier in the surgical pathway. In 2023, NHS England introduced a new programme where all hospitals were to implement early screening, triaging, risk assessment and health optimisation with the aim of increasing safety through reduced perioperative complications, increased efficiency (e.g., through reductions in last-minute cancellations), reductions in length of hospital stay, and identifying people who can undergo surgery without requiring an overnight stay. Here we describe the protocol to examine the implementation and impact of this new programme.

**Methods:**

A mixed methods research design will be used to evaluate this new programme. We will conduct a formative implementation evaluation using rapid qualitative methods consisting of interviews with staff members and patients across three broadly representative specialities (colorectal cancer surgery, repair of abdominal aorta and knee replacement) and observations of key meetings held regarding implementation activities. An interrupted time series analysis will address patient centered outcomes (days alive and out of hospital at 30, 180 and 365 days after surgery; postoperative hospital length of stay; emergency re-admission within 30 days) using routinely collected electronic health records.

**Discussion:**

This study will provide lessons on the barriers and facilitators to implementation and will highlight staff and patient experiences of the new programme. It will also assess impact on patient centred outcomes using routinely collected hospital data and quasi-experimental research methods.

## Introduction

Over 10 million operations are carried out every year in the UK, improving millions of lives [1]. While most operations are low risk, some result in patients having poor outcomes. Several challenges can lead to poor outcomes after surgery, many of which can be modified if detected early enough, and appropriately managed: examples include poor physical fitness (“deconditioning”), behavioural factors such as tobacco and alcohol dependence, and poorly managed individual comorbidities (e.g., diabetes, hypertension) [2]. These challenges can be compounded in patients with ‘multimorbidity’ – that is, where a patient has more than one long term condition. In the UK, half of all 65-year-olds have multimorbidity, and this gets more common amongst older patients and people living in deprived areas [3]. Patients with multimorbidity are less likely to be in the best condition to undergo an operation: they are therefore more likely to have their operation cancelled at short notice (because it might not be safe for them to have surgery), or have complications or poorer outcomes if they do have their operation [4,5]. This means that, at present, surgery provided by the NHS in England may not be as safe or as efficient as it could be.

Patient safety is a priority for healthcare systems. In safe systems, patients would be less likely to experience avoidable harm, both from their own conditions, and from the care and treatments provided to them. International estimates suggest that of 421 million hospitalisations annually, there are 42.7 million adverse events or unsafe experiences, making avoidable harm the 14th worldwide leading cause of death and serious illness [6]. Amongst the highest risk clinical settings are Surgical, Perioperative, Acute and Critical carE (SPACE) services. In the UK, SPACE services treat >25 million NHS patients annually [7,8], SPACE services are therefore, being rapidly redesigned to expand capacity and increase productivity, using largely untested pathways of care, some of which include the complex transition between primary and secondary care.

Surgery causes physiological stress [9]. For patients who are deconditioned, have poorly managed long-term conditions or multimorbidity, there is a higher risk of complications during and after surgery, which can impact patient outcomes for years after surgery [2,10]. Preoperative assessment evaluates a patient’s health prior to surgery. The aim of this assessment is to identify risks and where possible minimise them through optimisation - “the process of supporting and working with a patient to get their health in as good a state as possible before surgery” [11]. of any co-existing medical conditions. However, preoperative assessment often takes place close to the planned date of surgery, meaning there is little time to optimise the patients’ health. This can leave a difficult decision between a late notice cancellation of the operation or, in other cases, because the surgery is urgent proceeding with surgery that carries a greater risk of post-operative complications than if long-term health conditions had been optimised. Early preoperative screening is the process of evaluating a patient’s health earlier in the surgical pathway [11,12]. This process identifies health risks that may be modifiable through prehabilitation and optimisation and aims to improve the patient’s physical and or psychological condition in advance of surgery [11,12]. This may include the process of risk scoring and shared decision making. Early preoperative screening aims to lead to an optimisation plan that both the patient and surgical team agree with [12]. Potential benefits include better patient health outcomes and fewer short notice cancellations [3,5,13].

In 2023, all NHS hospitals in England were asked to implement a programme of early screening, triaging, risk assessment and health optimisation [11]. This included pre-assessment questionnaires and establishing an expanded perioperative care workforce. The directive was published in the 2023/24 NHS Standard Contract [14] followed by guidance published in May 2023 [11].These teams could consist of, for example, care co-ordinators, nurses and perioperative physicians who carry out assessments of health needs to identify surgical risk factors and inform pre- and post-operative care. The team would identify low-risk patients who do not need to attend face-to-face preoperative assessment and patients who could be treated in elective hubs focused on providing High Volume Low Complexity (HVLC) surgery. These services are intended to facilitate early shared decision making and optimisation of patient health, increasing patients’ suitability and readiness for surgery. A key anticipated benefit of these changes is increased safety through reduced perioperative complications (both in terms of complications from surgery, and the psychological harms associated with last-minute cancellations) [11]. The programme may also increase efficiency, e.g., through reductions in last-minute cancellations, reductions in length of hospital stay, and identifying people who can undergo surgery without requiring an overnight hospital stay [11].

The requirement for an early screening, triaging, risk assessment and health optimisation programme (hereinafter referred to as “the programme”) to be implemented was included in the NHS 2023/24 contract with the stipulation that the programme was to be implemented “no later than 31 March 2024”. This is a national programme, with implementation taking place across multiple NHS hospital organisations, in diverse geographic and socioeconomic contexts and within a variety of surgical pathways. It is likely that implementation progressed variably across different clinical and organisational settings. In addition, some settings or specialties (e.g., bariatric surgery) [15] have more well-established multidisciplinary preoperative pathways which aim to optimise comorbidities and reduce the risk of patients undergoing surgery without critical modifiable health issues having been addressed. In this manuscript we describe the mixed methods approach we will adopt to examine the implementation and impact of the new screening programme.

## Study aims and research questions

### Study aims

1. To evaluate staff, patient and carer experiences of the programme across NHS Trusts and factors influencing this, to generate lessons learnt from this programme and for future programmes of this kind.
  i) Explore patient experiences of the programme and their involvement in decision-making throughout the early screening and optimisation process.
  ii) Examine the perceptions of healthcare providers on the feasibility, benefits, and challenges of the programme.
2. Evaluate the effectiveness of early preoperative screening and optimisation
  i) Assess how early preoperative screening impacts patient outcomes, surgical complications, cancellation rates, and cost implications.
  ii) Determine the extent to which early screening influences patient readiness, overall health, and recovery outcomes.

### Research questions

Implementation evaluation

1. Programme design and implementation
  i) How was the programme designed, and what factors influenced this?
  ii) How are different NHS settings implementing the programme and to what extent have the activities been implemented?
  iii) What are the experiences of those implementing and delivering the programme?
  iv) What are the barriers and facilitators for different NHS sites in implementing the programme?
2. Patient experience
  i) What are patient perceptions and experiences of the preoperative optimisation process?
  ii) To what extent do patients feel informed and supported in shared decision-making regarding their surgical care?
  iii) Has the implementation of this programme resulted in changes to patient care as anticipated?

### Effectiveness evaluation

1. Patient Outcomes and Health Impact
  i) How does early preoperative screening impact rates of postoperative complications and patient recovery times?
  ii) What effect does early screening and optimisation have on rates of short-notice surgical cancellations?
  iii) To what extent does early identification and optimisation of health risks influence long-term health outcomes for patients?
2. Cost implications and Resource Allocation
  i) What are the cost implications of early preoperative screening and optimisation on healthcare resources and system efficiency?

## Methods

### Study design

A mixed methods research design will be used to evaluate this intervention. Specifically, we will a). conduct a formative implementation evaluation using rapid qualitative methods, and b) assess the programmes effectiveness via an interrupted time series analysis looking at patient centered outcomes using routinely collected electronic health records.

### Ethics approval and consent to participate

No data collection is required for quantitative analyses. Secondary analyses will be conducted on HES datasets. Ethical approval was obtained for the qualitative study as follows:

- For the component covering national and regional staff interviews: University College London Research Ethics Committee ID 26505/001.
- For the component covering staff based within NHS sites and patients: approved by the HRA and Health and Care Research Wales (HCRW), reference number: 24/NE/0109.

### Qualitative methodology: Implementation evaluation

This is to be a formative implementation evaluation of the programme as implemented nationally, across the NHS in England. It will use qualitative methods, including interviews with staff, patients and carers, meeting observations, and documentary analysis. The evaluation will be guided by the revised Consolidated Framework for Implementation Research (CFIR) [16] and the NICE patient experience framework [17]. CFIR is designed to aid understanding of factors influencing implementation and was recently extended to incorporate different types of outcome [16]. The NICE patient experience framework provides 5 standards to guide quality of care improvement.

### Qualitative methodology: Study timeline

- Months 1–2 – Implementation evaluation; gaining ethical and local research governance permissions.
- Months 3–13 – Implementation evaluation; recruitment, data collection and analysis will run in parallel. Interim findings disseminated.
- Month 14 – Implementation evaluation; final write-up of results and submission of outputs.

Participant recruitment for the implementation evaluation across all study sites is expected to begin on 1 March 2025 and is expected to be completed, along with data collection, by month 13.

### Qualitative methodology: Sampling

This implementation evaluation will be coproduced with academic researchers, national leadership, local services, and patient and carer representatives. It will employ rapid qualitative methods to enable ongoing dissemination of formative learning with participating national and local stakeholders. It will use a multiple-case study design. Case studies permit in-depth analysis of change and its implications within specific contexts, while cross-case comparison (i.e., comparison across multiple case sites) permits identification of both context-specific and cross-cutting lessons on implementation and impact.

To optimise learning, we will sample case sites purposively at organisational and service levels (Table 1) in a range of organisation types (e.g., NHS Foundation Trusts, Teaching Hospitals, District General hospitals), serving different populations in different settings (e.g., large urban area, more rural or mixed geographies). We will study implementation in three contrasting surgical ‘tracer conditions’, varying in terms of urgency/types of options available to patient and clinicians: [1] colorectal cancer surgery (a relatively common and potentially life-threatening surgical condition), [2] repair of abdominal aortic aneurism (prophylactic surgery to address significant risk), and [3] primary knee replacement (elective, non-urgent surgery to address long-term quality of life). Finally, we will sample services at different stages of implementation/uptake, to ensure that we can identify lessons both on common challenges and how services engage with these to progress implementation. NHSE England have provided us with a list of sites (who have consented to be contacted) and an indication of the degree of implementation across the different specialties. Degree of implementation will be one of the variables we consider (in addition to geographical region, type of hospital, etc.) to select the sites included in the study. This variation will help reduce the risk of bias in our sampling.

**Table 1:** Overview of sampling for service-level case studies.

Dimension	Characteristic	Examples
Organisational	Status	Secondary/tertiary referral centers Unit performs surgery only on local patients
	Population served/ Demographics	Age Gender Ethnicity Socioeconomic status
	Setting	Urban Rural/mixed
Tracer services	Urgency/nature of condition and surgical process	Colorectal cancer surgery: treating potentially deadly disease. Repair of abdominal aortic aneurism: prophylactic surgery to reduce likelihood of potentially fatal rupture. Knee replacement: elective, non-urgent surgery to address pain and enable physical wellbeing.
Implementation	Status/stage of progress	Substantial progress/uptake Limited progress/specific obstacles

Within cases, we will collect qualitative data from a range of sources to optimise our analysis of implementation, uptake, and outcome (Tables 2 and 3). We will interview stakeholders with different perspectives on new services, including people involved with organisation and delivery of early screening and optimisation and associated surgical care, service management and system oversight, and national programme leadership (Table 2). We will conduct non-participant observations of governance meetings (at service, region, and national level) related to safe and effective delivery of early perioperative screening and optimisation (Table 3). Finally, we will collect relevant documents, including project plans, service protocols, meeting minutes, service reviews, and information transfer/sharing policies to understand ongoing development of and decision-making around services. Data will be collected from 5 sites using the sampling framework outlined in Table 2. It is possible there will be a limited number of people with relevant experience of the new programme. Therefore, interviews will be completed once we either reach saturation, defined by no new data presenting in the interviews [18], or where all relevant participants have been approached.

**Table 2:** Overview of anticipated interviewees (NB numbers are ‘up to’/maximum).

	Total across all 5 sites
***Stakeholder group***	
Patients	25 (5 at each site)
Preoperative assessment teams	10 (2 at each site)
Surgical teams	15 (3 at each site)
Managers and administrators (team/hospital)	10 (2 at each site)
Regional context (e.g. primary/social care, ICS, charities)	20 (across the national/regional workforce)
***Total***	70

**Table 3:** Overview of anticipated non-participant observations (NB numbers are ‘up to’/maximum).

	Total across all 5 sites
***Activity***	
Oversight meetings - national	**4**
Oversight meetings - regional	**10**
Oversight meetings - service	**10**
*Total*	**24**

## Participants will be screened following the below inclusion and exclusion criteria

### Qualitative methodology: Inclusion criteria

Patients/carers – Interviews will be conducted with participants who:

- Have been offered surgery for one of the three tracer conditions in this study (colorectal cancer, abdominal aortic aneurysm, knee osteoarthritis – primary knee replacement), or care for someone who has been offered surgery for one of the three tracer conditions.
- Are aged 18 or over.
- Have capacity to consent to participation.
- Carers who are decision makers and wish to take part on behalf of the patients.
- Carers where the patient would like somebody present with them in the interview.

### Staff – Interviews and observations

- Working in the participating case sites and clinical settings.
- Preoperative assessment team members, including nurses and other non-medical professionals. Key roles will include nursing/non-medical lead; medical lead (usually an anaesthetist) and other medical preoperative assessment staff members. If applicable, non-registered healthcare workers (e.g., perioperative care coordinators; digital health assistants; healthcare assistants).
- Surgical teams (to include at least one surgeon, one surgical administrator, and one member of a booking and scheduling team).
- Managers (e.g., of team, division, and hospital).
- Wider context (e.g., regional system leaders, commissioners, patient representative groups).
• National programme leadership.

### Qualitative methodology: Exclusion criteria

All participants will be excluded if they are under 18 years of age or lack capacity to consent. Staff participants will be excluded if they have not been involved in organisation or delivery of the early perioperative screening and optimisation programme.

## Qualitative methodology: Recruitment

### Interviews

Potential staff interviewees will be identified through a combination of local documentation, identification by local stakeholders and engagement activities. Researchers will approach potential staff participants initially by sharing study information by email. Patient participants will be identified by local staff or research delivery teams. Patients who have accessed the clinical service under the new guidance may be approached in clinic, by email or phone. This might mean that local staff will share a copy of the PIS. Local staff or research delivery teams will gain written or verbal consent to pass on patient contact details in line with local R&D requirements. UCL researchers will then approach potential patient participants in line with the above sampling approach and inclusion and exclusion criteria. In line with good practice and UCL requirements, recruitment documentation (i.e., information and consent forms) will make clear the anticipated burden and risks of participating and emphasise the voluntary nature of participation. Should any participants find the interviews distressing as they recall uncomfortable events, we will offer appropriate support by referring participants to local or national wellbeing services, including General Practitioner (GP) services and they will be reminded that they can stop the interview at any time. All potential participants will be given at least 48 hours to decide whether they would like to take part in the interview. They will be asked to complete a consent form (written or electronic) and return it to the research team in advance of the interview and a mutually convenient time and platform (telephone or MS Teams) will be agreed upon.

### Non-participant observations

Key meetings for observation will be identified through a combination of local documentation, and identification by local stakeholders. Participant information sheets will be circulated with meeting papers to all attendees in advance of the meeting. Verbal agreement for the observation to take place will be requested from those taking part in the meeting and agreement will be documented in the minutes; confirmation of ongoing agreement to be observed will be obtained at the beginning of each event that is to be observed and recorded in the meeting minutes and observation fieldnotes.

## Qualitative methodology: Data collection

### Interviews

Staff and patient/carer interviews will be guided by semi-structured topic guides (see S1 File–S3 File. These will be co-developed with clinical and patient representatives and guided by our research questions. Participants will take part in a single interview lasting 30–60 minutes which will be recorded on MS Teams or digital recorders. During each interview, the researchers will take notes, and a summary of these notes will be added to the RREAL Sheet. We will collect the ethnicity of the participants to assess the diversity of respondents.

### Non-participant observations

Observations of key meetings will be conducted online. They will be guided by observation templates reflecting our research questions, e.g., exploring how these services are led and overseen, monitoring of uptake and impact of services, how decisions are made around emerging problems and opportunities to improve quality. Observations will be recorded as field notes collected by the researchers and saved on the secure UCL drive.

### Document review

Finally, Key documents will be either be in the public domain or will be documents not considered sensitive and will be shared with us by representatives of local services and the national programme.

### Qualitative methodology: Patient and public involvement

Two PPIE members have been recruited to join the implementation evaluation component of the study and are to be embedded throughout the full project lifecycle. Initial consultation with the PPIE members included a discussion of an outline of the study; its aims and objectives, and timelines, as well as a discussion around the proposed PPIE plan and if there were any training that the PPIE members felt they would benefit from to contribute to this study. PPIE members were given the opportunity to ask questions about the study and to contribute to the PPIE plan to ensure the activities were both reasonable and within their time capacity. PPIE members were remunerated following NIHR PPIE payment guidance [19]. Once a draft was finalised, the two members reviewed and provided feedback on this protocol, the participant information sheet, consent form and topic guides. In response to their feedback, we made amendments to the draft documents and provided responses to their feedback with what changes had or had not been made and why. Broadly, the study design was favourable with our PPIE members. However, one of the key changes we made to our protocol, following their feedback, was to restructure our aims and objectives so that they were easier to follow. We also discussed the importance and influence of the carer role in the patient pathway.

Throughout the study, PPIE members will be actively involved in discussions of our research approach and emerging findings; they will also contribute to discussions of the final analysis and implications of the research. In particular, these discussions will consider implications of the work for equality and diversity. If willing, the PPIE members will also be supported in contributing to dissemination activities, e.g., as co-authors of articles and summaries of findings, and speakers when presenting findings. PPIE members will be central to ensuring these outputs are presented in clear, accessible language, and that the lessons are relevant to patients and the public.

### Qualitative methodology: Data analysis

Data collection and analysis will be carried out in parallel and facilitated through the use of RREAL Sheets [20]. RREAL sheets will be developed per site (one for staff and one for patients) and used to facilitate cross-case comparisons and per population (to make comparisons between sub-groups). The RREAL sheets will then be used to summarise and share emerging findings on an ongoing basis [12]. Interview transcripts will be analysed using framework analysis; transcripts will be read for familiarisation; the research team will then label data considered relevant to the research questions (‘coding’). The codes will be grouped into categories to help form the final themes [21]. The framework will be informed by our initial research questions and topic guides, the main findings identified in the RREAL sheets and topics emerging from the data. The framework analysis for staff data will also be guided by the Consolidated Framework for Implementation Research (CFIR) [16] and the framework analysis for the patient data will be guided by the NICE patient experience framework [9]. The document review will facilitate the production of a programme theory to aid with our understanding of how the program is intended to work.

### Quantitative methodology: Effectiveness evaluation

### Data sources

For the effectiveness evaluation, we will use a cohort study research design using Hospital Episodes Statistics (HES) data. A bespoke dataset extracted from Hospital Episodes Statistics Admitted Patient Care (HES-APC), Critical Care (HES-CC) and HES linked Office for National Statistics (ONS) mortality data will be used to conduct this analysis.

HES is a high quality national secondary care database that reports all NHS hospital admissions in England. It includes detailed information on hospital activities including admission dates, diagnoses and procedures, duration of stay and patient demographic information [22]. The access to the data is managed by NHS Digital. Relevant data fields used in this research are detailed in S4 File.

### Quantitative data management

The effectiveness analysis will be conducted on pseudonymised data. Data linkage and extraction will be conducted by NHS Digital and once completed data will be held in a secure data system at UCL that meets ISO27001 certification [23]. All analyses will be conducted within the secure data system by appropriately trained researchers.

### Quantitative methodology: Study timeline

The expected start date for this aspect of the study will be 15 January 2026.

- Months 0–9 – Effectiveness evaluation; submit HES data access application, work to gain access to the linked dataset. While waiting for the data access, we will revise our statistical analysis plan.
- Months 10–12 – Effectiveness evaluation; data cleaning and exploratory data analysis. Interim findings disseminated via internal reports and presentations.
- Months 13–18 – Effectiveness evaluation; finalise statistical analysis, write-up and submission of outputs.

### Quantitative methodology: Primary outcome

The primary outcome of the quantitative study component is days alive and out of hospital (DAOH) at 30 days after surgery. This patient-centred outcome measure has been clinically validated internationally and is related to clinically relevant patient characteristics, surgical complexity, in-hospital complications, and long-term outcomes [24–26]. We will also assess DAOH at 180 and 365 postoperative days to provide information on longer term mortality outcomes [27].

We will use similar methodology to construct DAOH as detailed elsewhere [27,28]. Specifically, to calculate DAOH of a defined period, we will subtract the total post-operative duration of initial and any next subsequent hospital stays from the total period length to obtain the numbers of days spent out of hospital. The time spent in a non-hospital care facility will not be accounted for in our analyses [27]. DAOH will be defined as 0 days if a patient died within the defined period. A longer DAOH will be indicative of more favourable outcome. For each DAOH at 30, 180 and 365 variable respectively, there will be a ceiling 30/180/365. DAOH data will be calculated based on event date of corresponding operation procedure ICD-10 code, length of stay data related to each hospital admission and subsequent re-admission, discharge date and discharge destination from HES APC dataset and death date from HES-ONS linked mortality dataset.

### Quantitative methodology: Secondary outcomes

The secondary outcomes of this study are postoperative length of stay in hospitals and emergency re-admission within 30 days, as these are related to patient safety in peri-operative setting [29]. Both variables will be constructed using extracted data fields in HES data. In addition, we will examine days spent in critical care [30]. Finally, we will consider related costs using Healthcare Resource Groups (HRG) codes in HES datasets and linking them to the National Tariff.

In addition, we plan to assess the impact of early preoperative screening on the incidence of short-term cancellations by linking data from the Theatre Productivity Dataset with Perioperative Care Census conducted by NHS England [31]. These datasets collect information at the hospital site level, and are currently in their infancy. Data quality and completion will be assessed prior to any analyses to ensure findings are reliable and robust.

### Quantitative methodology: Effectiveness statistical analysis

The study population is defined to align with the qualitative component of the study. That is, all adults undergoing colorectal cancer surgery, repair of abdominal aortic aneurism or knee replacement at an NHS hospital in England between Jan 1^st^ 2018 and April 1^st^ 2026 in HES APC dataset.

In the absence of randomisation, quasi-experimental research designs are increasingly used for evaluating population health interventions [32,33]. In this analysis, we will use an interrupted times series research design: this method has been widely used to evaluate health interventions with time series data [34].

HES provides a long time series dataset, and, without a control group, a single interrupted time series analysis is the most suitable design to evaluate the programme [35]. We will conduct segmented regression analysis on monthly DAOH measures, postoperative length of stay, rate of emergency re-admission in 30 days,days spent in critical care and also relevant costs [36].

Based on date of surgery, in this analysis we will model monthly data at different time periods between Jan 1st 2018 to Jan 1st 2026, with Jan 2018 to end of Feb 2023 data as baseline period. Intervention period is March 2023 to end of March 2024. Post-intervention period is April 2024 to April 2026.

Specifically, we will model the following:


$$Y_{t}=\;\beta_{\text{0}}+\beta_{\text{1}}T+\beta_{\text{2}}X_{\text{1}t}+\beta_{\text{3}}{(T-T_{i})X}_{\text{1}t}\;+\beta_{\text{4}}X_{\text{2}t}+\beta_{\text{5}}{(T-T_{d})X}_{\text{2}t}\;+\;\varepsilon_{t}$$

Where Yt is the outcome at time t, T represents the time since the start of the study, X_1t_ is a dummy (0/1) variable indicating the intervention period [1] or otherwise 0, X_2t_ is a dummy variable representing the post-intervention period, Ti represents the time point when the intervention starts (X_1t_=1 for T ≥ Ti). Td represents the time point when the post-intervention period starts (X_2t_=1 for T ≥ Td). [37]

$\beta_{\text{0}}$ represents the baseline level of Y at T=0

$\beta_{\text{1}}$ represents underlying pre-intervention trend

$\beta_{\text{2}}$ represents the intercept change immediately following the intervention

$\beta_{\text{3}}$ represents the change in the slope of the trend following the intervention

$\beta_{\text{4}}$ represents the intercept change immediately post the intervention

$\beta_{\text{5}}$ represents the change in the slope of the trend post the intervention

Details of this methodology are available elsewhere [37].

In our analysis, by stratification, we will examine variations in time trend patterns in geographic location and patients characteristics including age groups, gender, ethnicity and deprivation measured using Index of Multiple Deprivation quantiles. We will use region of residence and local integrated care board in HES data to indicate geographical locations. Age will be grouped as 18–44, 45–65, 66–79, and 80 plus [38]. Ethnicity will be grouped based on ONS 2021 census ethnic categories, including White, Asian and Asian British, Black and Black British, Mixed or multiple ethnic groups, and Other ethnic group. In addition, we will explore variations in NHS hospital sites.

We will also check for seasonality impacts through sensitivity analyses such as using time stratified model, periodic functions or flexible spline functions that are detailed elsewhere [39]. The timing of rollout of the intervention was likely to be different across hospitals, therefore, we will conduct a sensitivity analysis with different definitions of the intervention period (i.e., early implementation period- 1^st^ March to 31^st^ Aug 2023 vs late implementation period – 1^st^ Sep 2023–31^st^ March 2024). Given the dramatic impact of Covid on health services, we will also assess the impact of a shorter baseline period of between Jan 2018 to Feb 2020. In addition, concurrent national programmes that target similar outcomes, such as Getting it Right First Time (GIRFT) [40], could confound our results. We will conduct sensitivity analyses that will stratify by different time periods to adjust for relevant national programmes if there are well-documented dates of implementation and can be distinctly modelled within our time series. We will conduct complete case analyses. Stata 17 (StataCorp) and R 4.2.2 (R Core Team, 2022) will be used to conduct the analysis.

Finally, to examine short-term cancellation, we will conduct descriptive data analysis and describe short-term cancellation data across different time periods and geographical regions.

### Integrated approach

The study will take a convergent design by carrying out a qualitative implementation evaluation alongside a quantitative effectiveness evaluation [41]. While the qualitative and quantitative analysis will be performed separately, the results will be presented using joint display tables to compare and relate data [41].

To better integrate our mixed-methods approach, we will utilise findings from our qualitative implementation evaluation to inform our quantitative analysis. Our qualitative study will provide rich and contextual understanding of implementation barriers and facilitators. While it is not possible to do this for all hospitals in HES dataset, we will develop a typology of implementation. This will allow us to categorise the measured level and success of implementation of the programme from five study sites and then conduct a quantitative case study analysis to explore the relationship between the measured success/maturity of implementation and patient level outcomes, and will facilitate a more nuanced interpretation of the national findings.

## Discussion

Early screening, triaging, risk assessment and health optimisation in perioperative pathways is an important programme built on evidence that may lead to improvement in patient outcomes and NHS efficiency. This mixed methods study will explore staff and patient experiences and perceptions, as well as assess the impact of the programme on patient centred outcomes using routinely collected hospital data and quasi-experimental research methods.

The implementation evaluation will be carried out across multiple NHS Regions providing preliminary findings as the evaluation is ongoing. This will facilitate real time insights, delivered to stakeholders through formative feedback, allowing us to explore the barriers and facilitators as they happen and at different stages of implementation as some sites will be further developed than others. This process of providing formative feedback will offer the opportunity for stakeholders to further improve implementation strategies through real time adaptation [42]. Another key strength of this research is that we are able to use data from all hospitals in England to evaluate a range of patient level outcomes and cost analysis.

The quantitative effectiveness evaluation has several important limitations. First, the study lacks a randomised control group because the early screening programme was implemented nationally across England in all specialties and we only have data for hospitals in England. Without a control group, this makes a single interrupted time series analysis the most suitable approach. Second, the analysis cannot definitively link the intervention to individual patients, relying instead on the timing of the programme’s implementation. This is due to the level of uncertainties around exact timing of the programme’s rollout and differences in pre-existing established early screening and optimisation services across different hospitals in the country. To deal with this, we will conduct several sensitivity analyses using different start dates for the intervention period to account for both early and late implementation and also stratify our analyses by regions or hospitals using results from our short-term cancellation data and qualitative study. Third, this design is susceptible to confounding from co-occurring events and policy changes in the same period. To address confounding events such as in the case of COVID, we will be conducting a sensitivity analysis using a shorter pre-COVID baseline period and by using our qualitative findings to provide context for any observed changes. Fourth, unmeasured confounding may also bias our results. Fifth, the analysis relies on routinely collected hospital data, which may have data quality concerns [43]. To address this, we will conduct thorough data cleaning and preparation to ensure our findings are robust.

We acknowledge that programme success is more complex than simple statistical improvements. It is possible that the programme’s success is demonstrated by widening access to surgery for higher-risk patients who would have been previously excluded, which might result in static or even worsened postoperative complication rates and lengths of stay. To account for this, we will also monitor changes in underlying patient characteristics, such as the percentage of patients in the most deprived quintile of the Index of Multiple Deprivation, which is strongly correlated with multimorbidity rates [44].

Table 4 provides a summary outlining the key components of the study.

**Table 4:** Summary Table outlining key components of the study.

	Qualitative	Quantitative
Research questions	Programme design and implementation 1. How was the programme designed, and what factors influenced this? 2. How are different NHS settings implementing the programme and to what extent are the activities implemented? 3. What are the experiences of those implementing and delivering the programme? 4. What are the barriers and facilitators for different NHS sites in implementing the programme? Patient experience 1. What are patient perceptions and experiences of the preoperative optimisation process? 2. To what extent do patients feel informed and supported in shared decision-making regarding their surgical care? 3. Has the implementation of this programme resulted in changes to patient care as anticipated?	Patient Outcomes and Health Impact 1. How does early preoperative screening impact rates of postoperative complications and patient recovery times? 2. What effect does early screening and optimisation have on rates of short-notice surgical cancellations? 3. To what extent does early identification and optimisation of health risks influence long-term health outcomes for patients? Cost implications and Resource Allocation 1. What are the cost implications of early preoperative screening and optimisation on healthcare resources and system efficiency?
Timelines	Months 1–2 – Implementation evaluation; gaining ethical and local research governance permissions. Months 3–13 – Implementation evaluation; recruitment, data collection and analysis will run in parallel. Interim findings disseminated. Should any sites experience delays in implementation, the data collection period will be extended. Month 14 – Implementation evaluation; final write-up and submission of outputs. Participant recruitment for the implementation evaluation has begun.	Months 0–9 – Effectiveness evaluation; submit HES data access application, work to gain access to the linked dataset. While waiting for the data access, we will revise our statistical analysis plan. Months 10–12 – Effectiveness evaluation; data cleaning and exploratory data analysis. Interim findings disseminated via internal reports and presentations. Months 13–18 – Effectiveness evaluation; finalise statistical analysis, write-up and submission of outputs.
Data sources	Qualitative implementation evaluation: • Staff and patient interviews • Non-participant observations • Document review	Quantitative effectiveness evaluation: • Hospital Episodes Statistics (HES)

## Supporting information

S1 File Patient interview topic guide. (DOCX)

S2 File Regional staff interview topic guide. (DOCX)

S3 File Service staff interview topic guide. (DOCX)

S4 File HES and HES linked ONS data fields. (DOCX)

## Abbreviations

DSH: UCL Data Safe Haven
GDPR: General Data Protection Regulation
GP: General Practice
HES: Hospital Episode Statistics
HES-APC: Hospital Episode Statistics- Admitted Patient Care
HES-CC: Hospital Episode Statistics- Critical Care
ONS: Office for National Statistics
NHS: National Health Service
PIS: Participant Information Sheet
PPIE: Patient and Public Involvement and Engagement
PSRC: NIHR Central London Patient Safety Research Collaboration
REC: Research Ethics Committee
REDCap: UCL Research Data Collection Service
UCL: University College London
