# Supplementary material for: A mixed methods study protocol evaluating early screening, triaging, risk assessment and health optimisation in perioperative pathways
Source: PLoS One. 2025 Nov 5;20(11):e0335129. doi: 10.1371/journal.pone.0335129 (PMC12588520; doi:10.1371/journal.pone.0335129)
Supplement: S1 File — (DOCX) [file pone.0335129.s001.docx]

**PSRC – E-PERISCOPE – Patient study**

*Interview topic guide*

**Experience of diagnosis & decision-making**

- Can you tell me about how you obtained your diagnosis and treatment plan?
- How long after the diagnosis did you decide to have surgery?
- When was this decision made?
- Were you part of the decision to go ahead with surgery, and if so, how?
  - Were treatments other than surgery discussed with you?
  - Was the option not to have surgery discussed with you?
  - Do you think you could have been involved more in this decision, and if so, how?

**Information**

- Who or what helped you with making the decision about whether to have surgery? (e.g. hospital staff, family, friends, other sources of information and advice)
- Were you provided with relevant new information to help you make your decision? If so, how were you provided with this information (e.g. in person, printed information, website, app)?
  - Did the information provided reflect any additional communication needs you have?
  - Did the information provided feel personal to you?
  - Did it account for any current health issues you have, as well as your background more generally?
- Did you feel you had enough time to consider your options around surgery and come to a decision on how to proceed?

**Communication**

- Have you been given a date for your operation? If you have, when were you given this date?
- What information were you provided with from the healthcare providers around what to expect before surgery and the recovery process?
- When do you recall first being asked by a member of the hospital team about your health more generally as part of the process of planning for surgery?
- How did you provide this information (e.g. in person, over telephone, paper form, web form, app)?
- Did you receive advice or guidance on improving your health prior to your operation?
  - If so, did you feel this advice took account of your circumstances? (e.g. time available, financial resources)
- How frequently has someone been in touch with you to confirm whether there have been any changes in your health since deciding to proceed with surgery?
  - How have the surgical team kept in contact with you (e.g. text messages, phone calls, emails, letters, in person appointments)?
  - Do you know who you should speak to if you had questions about your operation?
- Do you know if any of the questions you were asked about your health could have been answered by the GP or another primary health care professional instead?

**Support**

- What help were you provided with during the surgery and recovery process?
  - What elements were most helpful?
  - Overall, did you feel well supported by the healthcare system?
- Did you seek, or were you provided, support from any provider outside of the healthcare system prior to your surgery?
- Did you have any carers (family/friends) supporting you through this process and if so, what role did they play?
- How did this experience impact you emotionally, physically, financially?
  - Do you feel you have been treated with dignity and respect?
- Have you felt safe during this process?

**Reflections**

- Is there anything you wished you had known or had access to when you were going through surgery and recovery?
- Is there anything you wished you had known or had access to during this process?
