# Supplementary material for: A mixed methods study protocol evaluating early screening, triaging, risk assessment and health optimisation in perioperative pathways
Source: PLoS One. 2025 Nov 5;20(11):e0335129. doi: 10.1371/journal.pone.0335129 (PMC12588520; doi:10.1371/journal.pone.0335129)
Supplement: S2 File — (DOCX) [file pone.0335129.s002.docx]

**PSRC – E-PERISCOPE**

**Interview topic guide: Regional level**

**Background**

1. Please tell me about your background, role, and how long you have been in this role
2. How are you involved in implementing early screening, risk assessment and health optimisation in your region?
3. What do you think are the drivers/purpose behind the initiative?
4. What did you think of the idea? What did your colleagues think?
5. What is your impression of how the national programme team have worked with your region on this?

*For regional leads, or others involved in policy development:*

1. How was the programme, designed and developed? Who was involved in this process?
2. What factors influenced this approach?
3. Was a particular programme theory used?
4. How were local contexts considered in the design of the policy?
5. Geography and organisational status?
6. Clinical conditions and pathways?
7. Equity of access (e.g. health literacy/patient activation, socioeconomic status, language, caring responsibilities, disability)?
8. Stage of digitalisation? (Both for information sharing between primary and secondary care, and digitally-enabled perioperative assessment)
9. How was the perioperative care workforce strategy, including the non-clinical perioperative care coordinator role, developed?
10. What leaders, opinion formers, and other supporters were involved?

**Governance and management**

1. Can you describe how the updated pathway works and who is involved at each stage?
2. How is implementation being managed and supported at a regional level?
3. Processes (e.g. meetings, data collection)
4. Who is involved? (e.g. clinicians, patients and carers, voluntary sector, politicians)
5. What factors influenced this approach?
6. What resources are required? (time, money, workforce)
7. What other support has been provided to service level teams? (e.g. funds, guidance, training, engagement from national leadership)
8. How do regional leads communicate and work together with both national and service level leaders?
9. Who is involved? (e.g. national/local teams, integrated care systems, commissioners, committees, patients, carers, voluntary sector, politicians)
10. How do you keep up with national and local developments?
11. How have regions been supported from a national level? (e.g. funds, guidance, training, engagement from national leadership)
12. If known, how is the new service governed within hospitals?
13. What level of oversight do regions have?
14. Why were these approaches taken?
15. What has the reception been?
16. What are the challenges?
17. How do different services within the system work together? (including perioperative care teams, surgical teams, primary care, other community actors)

**Implementation progress**

1. How are you measuring progress of local implementation?
2. Processes (reviewing data, meetings, focus groups, surveys)
3. Focus groups (milestones, hospital/service level, learning)
4. How is the implementation progressing? (including uptake, fidelity, adaptation)
5. Are there any geographical or clinical areas that are further developed than others?
6. Are there any geographical or clinical areas that are less developed than others?
7. Which factors have influenced this?

*For both questions 14 and 15*

1. Finance
2. Leadership
3. Staffing (particularly recruitment to the non-clinical perioperative care coordinator role)
4. Technology (particularly interoperability between primary and secondary care, and platforms for digitally-enabled perioperative assessment)
5. How might differences in uptake be influencing perioperative outcomes?

**Current and future impact**

1. What factors have facilitated the implementation?
2. Do you foresee any factors that have or may prevent or delay the implementation?
3. How do you think the service will impact staff and patients?
4. Can you think of any unintended consequences of the implementation?
5. Patient safety/outcomes (complications, length of stay, cancellations)
6. Quality of care
7. Patient experience
8. Equity of access for underserved communities (e.g. geography, culture, language, education, technological capability)
9. Workforce (experience, sustainability)
10. Costs and resource use (including staffing)
11. Overall, how would you sum up your experience of implementation of this policy at the regional level?
12. Is there anything you think needs to be done differently?
13. What is the potential for further development or lessons for future implementation?
14. Is there anything you would like to add that we haven’t already talked about?
