# Supplementary material for: A mixed methods study protocol evaluating early screening, triaging, risk assessment and health optimisation in perioperative pathways
Source: PLoS One. 2025 Nov 5;20(11):e0335129. doi: 10.1371/journal.pone.0335129 (PMC12588520; doi:10.1371/journal.pone.0335129)
Supplement: S3 File — (DOCX) [file pone.0335129.s003.docx]

**PSRC – E-PERISCOPE**

**Interview topic guide: Service level**

**Background**

1. Please tell me about your background, role, and how long you have been in this role
2. How are you involved in implementing early screening, risk assessment and health optimisation in your service area?
3. Specific activities, time allocated
4. Are you clear on your role within this service?
5. How would you describe the drivers/purpose of the new policy?
6. What did you think of the idea? What did your colleagues think?

**Governance and management**

*This section may not apply to all staff*

1. Can you describe how the updated pathway works and who is involved at each stage?
2. How is implementation being led in your team/organisation?
3. People
4. Which professional roles have been involved in the implementation of early screening and health optimisation?
5. What leadership is in place?
6. Would the involvement of any other roles/personnel be helpful in improving the success of implementation?
7. Processes
8. What processes are in place to support implementation? (e.g. planning meetings, governance)
9. What are the factors that have led to early screening being implemented in some surgical specialities/pathways and not others?
10. Measures
11. How is progress of implementation measured and recorded? (e.g. extent/spread of uptake, and whether people are following recommended process)
12. How has this worked? Anything that could be done differently?
13. How do you hear about progress with implementation?
14. Do you know how national and regional stakeholders are being kept up to date with developments?
15. What support have sites received from a regional and national level? (Funds, guidance, training, engagement from regional leadership, other)
16. Has this support been sufficient?

**Implementation progress**

1. How long did the implementation take to set up and get to the current stage?
2. What stage is implementation at regarding early screening after referral for surgery?
3. What stage is implementation at with respect to the development of personalised support plans for patients requiring health optimisation?
4. What stage is implementation at with respect to maintaining contact with patients on waiting lists at least every three months?
5. What stage is implementation at with respect to facilitating shared decision-making conversations?
6. How has the service been reconfigured to support these new requirements?
7. How have things changed in terms of how your work interacts with other areas of the hospital?
8. Processes (patient pathway, protocols, SOPs)
9. Staffing (numbers, rota) – has staffing changed to support the implementation or the new process going forward?
10. New roles (specialised roles, promotion or change of role – in particular the non-clinical perioperative care coordinator)
11. Support – training, guidance, technology (particularly to enable interoperability between primary and secondary care, and platforms for digitally-enabled perioperative assessment)?
12. Of what kind? Was it useful?
13. Would any other support be helpful?
14. How is the new policy being led and delivered?
15. Does it work well?
16. Which factors are influencing this?
17. Has your collaboration with primary care changed at all as a result of this work?
18. What factors are acting as barriers or facilitators to the implementation?

**Current and future impact**

1. Has the programme produced an impact on the way in which services are delivered?
2. How is the service measuring impact? (e.g. waiting list length, length of stay, rate of surgical complications, costs and cost-effectiveness)
3. How are patients finding the early screening and potential health optimisation?
4. Is the new service beneficial to patients?
5. Is it improving safety outcomes?
6. How do you think the new service is affecting staff?
7. Are you aware of any unintended consequences of the programme at present?
8. Patient safety/outcomes (complications, length of stay, cancellations)
9. Quality of care
10. Patient experience
11. Equity of access for underserved communities (e.g. geography, culture, language, education, technological capability)
12. Workforce/staff – experience, sustainability
13. Costs and resource use (including staffing)
14. Overall, how would you sum up the implementation in this organisation?
15. Is there anything you think needs to be done differently?
16. What is the potential for further development or lessons for future implementation?
17. Is there anything you would like to add that we haven’t already talked about?
