# Supplementary material for: A mixed methods study protocol evaluating early screening, triaging, risk assessment and health optimisation in perioperative pathways
Source: PLoS One. 2025 Nov 5;20(11):e0335129. doi: 10.1371/journal.pone.0335129 (PMC12588520; doi:10.1371/journal.pone.0335129)
Supplement: S4 File — (DOCX) [file pone.0335129.s004.docx]

## Supplementary Information

**S1 HES and HES linked ONS data fields**

| **HES APC Field** | **Field names** |
| --- | --- |
| ADMIAGE | Age on Admission |
| ADMIDATE | Admission Date (Hospital Provider Spell) |
| ADMIMETH | Method of Admission |
| ADMINCAT | Administrative Category Code on Admission |
| ADMISORC | Source of Admission |
| BEDYEAR | Bed Days Within the Year |
| CLASSPAT | Patient Classification |
| CR_TREATMENT | Commissioning Region of Treatment |
| GPPRAC | General Medical Practice – patient registration |
| CR_RESIDENCE | Commissioning Region of Residence |
| CURRWARD_ONS | Current Electoral Ward (ONS) |
| DIAG_3_CONCAT | Concatenated Diagnosis Codes - 3 Character |
| DIAG_3_nn | Diagnosis Code - 3 Character |
| DIAG_4_CONCAT | Concatenated Diagnosis Codes - 4 Character |
| DIAG_4_nn | Diagnosis Code - 4 Character |
| DIAG_COUNT | Count of Diagnoses |
| DIAG_nn | Diagnosis Code |
| DISDATE | Date of Discharge |
| DISDEST | Destination on Discharge |
| DISMETH | Method of Discharge |
| ELECDUR | Duration of Elective Wait (Derived) |
| ELECDUR_CALC | Duration of Elective Wait (Calculation) |
| EPIDUR | Episode Duration |
| EPIEND | Episode End Date |
| EPIKEY | HES Record Identifier |
| EPIORDER | Episode Order |
| EPISTART | Episode Start Date |
| EPISTAT | Episode Status |
| EPITYPE | Episode Type |
| ETHNOS | Ethnic Category |
| FAE | Finished Admission Episode flag |
| FAE_EMERGENCY | Finished Admission Episode, Emergency Classification flag |
| FCE | Finished Consultant Episode flag |
| FDE | Finished Discharge Episode flag |
| IMD04 | IMD Index of Multiple Deprivation |
| IMD04_DECILE | IMD Decile Group |
| IMD04C | IMD Crime Domain |
| IMD04ED | IMD Education Training and Skills Domain |
| IMD04EM | IMD Employment Deprivation Domain |
| IMD04HD | IMD Health and Disability Domain |
| IMD04HS | IMD Barriers to Housing and Services Domain |
| IMD04I | IMD Income Domain |
| IMD04IA | IMD Income Affecting Adults Domain |
| IMD04IC | IMD Income Affecting Children Domain |
| IMD04LE | IMD Living Environment Domain |
| IMD04RK | IMD Overall Rank |
| LSOA11 | Lower Super Output Area of Residence 2011 |
| MAINSPEF | Main Specialty |
| OPDATE_nn | Date of Procedure |
| OPERSTAT | Operation Status Code |
| OPERTN_3_CONCAT | Concatenated Procedure Codes - 3 Character |
| OPERTN_3_nn | Procedure Code - 3 Character |
| OPERTN_4_CONCAT | Concatenated Procedure Codes - 4 Character |
| OPERTN_4_nn | Procedure Code - 4 Character |
| OPERTN_COUNT | Count of Procedures |
| OPERTN_DATE | Date of Procedure |
| OPERTN_nn | Procedure Code |
| POSOPDUR | Post-Operative Duration |
| PSEUDO_HESID | Pseudonymised Patient identifier - HES generated |
| SEX | Person Stated Gender Code |
| SPELBGIN | Beginning of Spell Indicator |
| SPELDUR | Duration of Spell |
| SPELEND | End of Spell Indicator |
| SUSHRG | Healthcare Resource Group - SUS Generated |
| TRETSPEF | Treatment Specialty |
| **HES Critical Care (CC) Field** |  |
| ACARDSUPDAYS | Advanced Cardiovascular Support Days |
| ARESSUPDAYS | Advanced Respiratory Support Days |
| BCARDSUPDAYS | Basic Cardiovascular Support Days |
| BESTMATCH | Best Match Flag |
| BRESSUPDAYS | Basic Respiratory Support Days |
| CCADMISORC | Critical Care Admission Source |
| CCADMITYPE | Critical Care Admission Type |
| CCDISDATE | Critical Care Discharge Date |
| CCDISDEST | Critical Care Discharge Destination |
| CCDISLOC | Critical Care Discharge Location |
| CCLEV2DAYS | Critical Care Level 2 Days |
| CCLEV3DAYS | Critical Care Level 3 Days |
| CCSTARTDATE | Critical Care Start Date |
| CCSTARTTIME | Critical Care Start Time |
| LIVERSUPDAYS | Liver Support Days |
| NEUROSUPDAYS | Neurological Support Days |
| ORGSUPMAX | Organ Support Maximum |
| PSEUDO_HESID | Pseudonymised Patient identifier - HES generated |
| RENSUPDAYS | Renal Support Days |
| **ONS linked secondary care mortality Field** |  |
| PSEUDO_HESID | Pseudonymised Patient identifier - HES generated |
| DOD | Date of Death |
